# Identifying Niemann–Pick type C in early-onset ataxia: two quick clinical screening tools

**DOI:** 10.1007/s00415-016-8178-0

**Published:** 2016-06-17

**Authors:** Matthis Synofzik, Zofia Fleszar, Ludger Schöls, Jennifer Just, Peter Bauer, Juan V. Torres Martin, Stefan Kolb

**Affiliations:** 1 Department of Neurodegenerative Diseases, Hertie-Institute for Clinical Brain Research, University of Tübingen, Hoppe-Seyler-Str. 3, 72076 Tübingen, Germany; 2 German Research Center for Neurodegenerative Diseases (DZNE), Tübingen, Germany; 3 Institute of Medical Genetics and Applied Genomics, University of Tübingen, Tübingen, Germany; 4 Syntax for Science SL, Basel, Switzerland; 5 Actelion Pharmaceuticals Ltd, Allschwil, Switzerland

**Keywords:** Niemann–Pick disease type C, Early onset ataxia, Suspicion index, Diagnosis

## Abstract

Niemann–Pick disease type C (NP-C) is a rare multisystemic lysosomal disorder which, albeit treatable, is still starkly underdiagnosed. As NP-C features early onset ataxia (EOA) in 85–90 % of cases, EOA presents a promising target group for undiagnosed NP-C patients. Here, we assessed the ability of the previously established NP-C suspicion index (SI) and a novel abbreviated ‘2/3 SI’ tool for rapid appraisal of suspected NP-C in unexplained EOA. This was a retrospective observational study comparing ‘NP-C EOA’ cases (EOA patients with confirmed NP-C) with non-NP-C EOA controls (EOA patients negative for NP-C gene mutations). NP-C risk prediction scores (RPS) from both the original and 2/3 SIs were calculated and their discriminatory performance evaluated. Among 133 patients (47 NP-C EOA cases; 86 non-NP-C EOA controls), moderate (40–69 points) and high (≥70 points) RPS were common based on original SI assessments in non-NP-C EOA controls [16 (19 %) and 8 (9 %), respectively], but scores ≥70 points were far more frequent [46 (98 %)] among NP-C EOA cases. RPS cut-off values provided 98 % sensitivity and 91 % specificity for NP-C at 70-point cut-off, and ROC analysis revealed an AUC of 0.982. Using the 2/3 SI, 90 % of NP-C EOA cases had scores of 2 or 3, and RPS analysis showed an AUC of 0.961. In conclusion, the NP-C SI and the new, quick-to-apply 2/3 SI distinguished well between NP-C and non-NP-C patients, even in EOA populations with high background levels of broadly NPC-compatible multisystemic disease features. While the original SI showed the greatest sensitivity, both tools reliably aided identification of patients with unexplained EOA who warranted further investigation for NP-C.

**Electronic supplementary material:**

The online version of this article (doi:10.1007/s00415-016-8178-0) contains supplementary material, which is available to authorized users.

## Introduction

Early onset ataxias (EOAs) present a highly heterogeneous group of degenerative and metabolic diseases, predominantly caused by recessive mutations in genes with pleiotropic, multisystemic manifestations [8]. A genetic diagnosis can be found in approximately 55 % of cases in European cohorts [2], and in up to 66 % cases in non-European cohorts with high consanguinity [5].

The range of ataxia disorders with identified genetic and phenotypic heterogeneity has been expanding rapidly in recent years, complicating and prolonging the diagnostic process in patients presenting with unclear EOA [8]. However, given the stark absence of therapies in this disease group, confirming a diagnosis is particularly important in cases where there is an evidence-based drug treatment available that can treat the underlying disease.

Niemann-Pick disease type C (NP-C) is a rare autosomal recessive lysosomal lipid storage disorder featuring a wide variety of visceral, neurological and psychiatric manifestations [13, 16], with EOA as a cardinal feature: 85–90 % of all NP-C cases present with EOA [10, 13]. An effective disease-modifying drug treatment for this condition is available [1, 9]. However, a large proportion of adult NP-C patients still remain undiagnosed [14], which emphasises the need for easy-to-apply clinical tools that can help identify treatable patients among those with as yet unexplained EOA. In a previous study, targeted high-throughput genetic screening identified NP-C diagnoses in a total of 6/204 (2.9 %) consecutive EOA patients [12]. A clinical and laboratory diagnostic study in 24 patients with both EOA and pre-senile cognitive decline resulted in NP-C diagnoses in four patients (17 %) [10]. Such findings indicate that the clinical EOA population is substantially enriched for NP-C.

The NP-C Suspicion Index (SI; http://www.npc-si.com) is an online clinical screening tool that was developed to help identify patients who may require further in-depth laboratory investigation for NP-C [15]. However, the discriminatory performance of the NP-C SI in patient groups with high levels of NPC-compatible disease features, in particular those with EOA, has been questioned [3, 10].

Here, we conducted a large retrospective cohort study aiming to determine whether: (1) the previously established NP-C SI indeed allows discrimination between EOA patients with NP-C (NP-C EOA cases) and EOA patients without NP-C (EOA controls) and (2) a novel abbreviated ‘2/3 SI’ might allow an even more rapid appraisal of suspected NP-C in unexplained EOA.

## Methods

### Patients

The EOA control group comprised a consecutive series of 86 index patients with early onset degenerative ataxia (age at onset <40 years) and a family history consistent with autosomal recessive inheritance (no ataxia in the parental generation), and who were tested negative for NP-C gene mutations, recruited between 2006 and 2012. *NPC1* and *NPC2* mutations were ruled out as part of a high-coverage (>94 % with a depth ≥20) custom-built targeted resequencing HaloPlex gene panel (Agilent, Santa Clara, CA, USA), which included 122 known ataxia genes (see Synofzik et al. [12] for details).

NP-C EOA cases comprised a multicentre cohort of patients assessed in five centres in Europe and Australia between July 2010 and January 2011, who had EOA (i.e. degenerative ataxia with age of onset <40 years) and confirmed NP-C diagnosed in the clinical practice setting based on filipin staining as well as *NPC1/NPC2* mutation analysis. All patients included in this analysis were aged >4 years.

### Assessments

Clinical signs and symptoms were evaluated by retrospective chart review in NP-C EOA cases and EOA controls by systematic phenotyping according to the standard SI protocol [15]. For reasons of simplicity and conformity, we continued to use the term ‘vertical supranuclear gaze palsy (VSGP)’ from this SI protocol, although this type of central oculomotor disorder is more appropriately described as ‘vertical supranuclear *saccade* palsy’. The discriminatory performance of the NP-C SI was subsequently compared in these two patient groups based on calculated SI risk prediction scores (RPS), with appropriate sensitivity and specificity analyses.

To provide a very brief tool for rapid clinical appraisal, analyses were conducted to investigate a new, simplified version of the original SI. Sensitivity/specificity analyses were performed based on univariable logistic regression for 19 signs and symptoms plus the ‘sibling with NP-C or cousin with NP-C’ item included in the original SI tool [15]. The three signs and symptoms that provided the greatest sensitivity and specificity were selected for the development of a ‘2/3’ SI model, which attributed one point for the presence of each of the three key manifestations in combination with ataxia.

### Data analysis

Patient demographics, disease manifestations and RPS scores were summarised using descriptive statistics. Between-group statistical comparisons of descriptive data on demographics and disease manifestations in NP-C EOA cases and EOA controls were conducted using the Wilcoxon Mann–Whitney for continuous data, and Chi-square or Fisher’s exact tests where applicable for categorical data.

For assessment of both the original SI and the new 2/3 SI tool in all ataxic patients, the relationship between the calculated RPS and the likelihood of NP-C was modelled using univariable logistic regression (ULR) as described previously [15]. Logistic regression modelling was performed using Proc Logistic in SAS version 9.3.

Discriminatory performance was assessed based on receiver operating characteristic (ROC) curves and subsequent area under the curve (AUC) analyses. Sensitivity and specificity values were plotted versus total RPS and tabulated at fine RPS intervals to assess the performance for different cut-offs.

## Results

### Patients

A total of 133 patients were included in the study: 47 NP-C EOA cases and 86 EOA controls (Table 1). The proportions of female patients in the NP-C EOA case and EOA control groups were similar (49 and 44 %, respectively). The mean (SD; range) age of NP-C EOA cases was lower [24 (13) years; 4–52] than that in the EOA control group [31 (12) years; 7–62].

**Table 1:** Patient demographics

	NP-C EOA cases (*N* = 47)	EOA controls (*N* = 86)
Gender, n (%) female^a^	23 (49)	38 (44)
Age (years)^b^		
Mean (SD) age	24 (13)	31 (12)
Median (range)	21 (4–52)	32 (7–62)

Statistical comparisons (NP-C vs. non-NP-C EOA cases)

^a^Chi-square test, *p* = 0.599

^b^Wilcoxon-Mann–Whitney test, *p* = 0.001

In the EOA control group, 35/86 (41 %) of the index EOA cases remained genetically unexplained after screening for the panel of 122 known ataxia-related gene mutations, while a molecular diagnosis for causal ataxia gene mutations was established in 51/86 (59 %) cases (Supplementary Table 1).

### Signs and symptoms: descriptive data

**Fig. 1:**
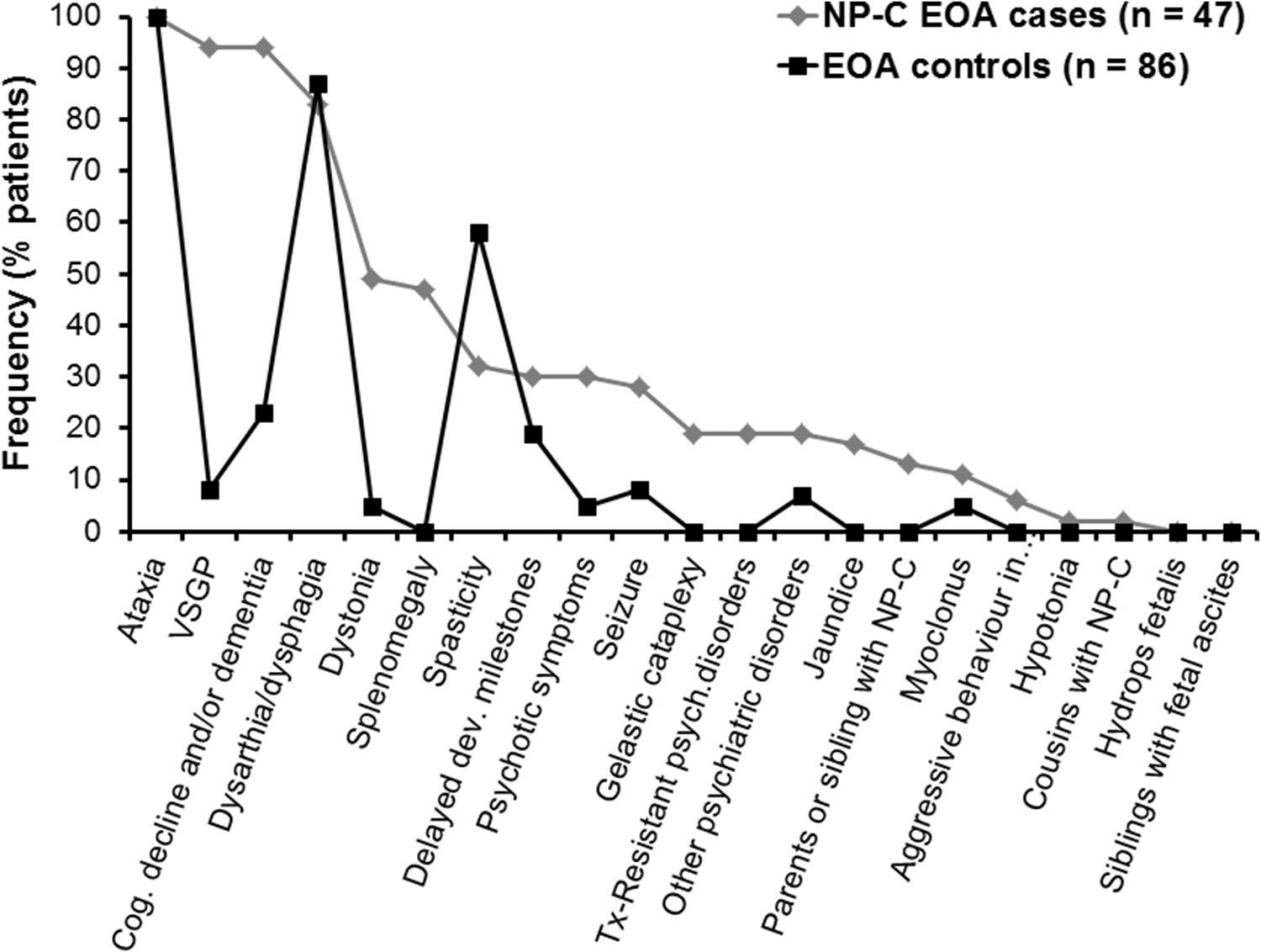
Symptom frequency in NP-C EOA cases versus EOA controls

The frequencies of NP-C signs and symptoms included in the original NP-C SI tool [15] in both EOA groups are summarised in Fig. 1. Among neurological manifestations, VSGP, cognitive decline/dementia and dysarthria/dysphagia were all recorded in association with ataxia in >80 % of NP-C EOA cases. Among EOA controls, the most common neurological manifestations (observed in >50 % patients) were dysarthria/dysphagia (in 87 % patients) and spasticity (in 58 %).

Descriptive data analysis allowed identification of the most frequent NP-C disease manifestations that occurred substantially more frequently in NP-C EOA cases compared with EOA controls, and which could therefore potentially serve as differentiating features between these two specific patient groups. These included VSGP (in 94 % of NP-C EOA cases versus 8 % of EOA controls; *p* < 0.001), cognitive decline and/or dementia (94 versus 23 %, respectively; *p* < 0.001), splenomegaly (47 versus 0 %; *p* < 0.001), dystonia (49 versus 5 %; *p* < 0.001), any psychotic signs other than cognitive decline/dementia (6–30 versus 7 %; *p* values 0.101 to <0.001), and seizures (28 versus 8 %; *p* = 0.004).

Notably, dysarthria/dysphagia and delayed developmental milestones occurred in similar proportions of patients in the two patient groups. ‘Acquired and progressive spasticity’ was more common in the general EOA population compared with NP-C EOA cases (58 versus 32 %, respectively; *p* = 0.006).

### Assessment of original NP-C SI performance

**Fig. 2:**
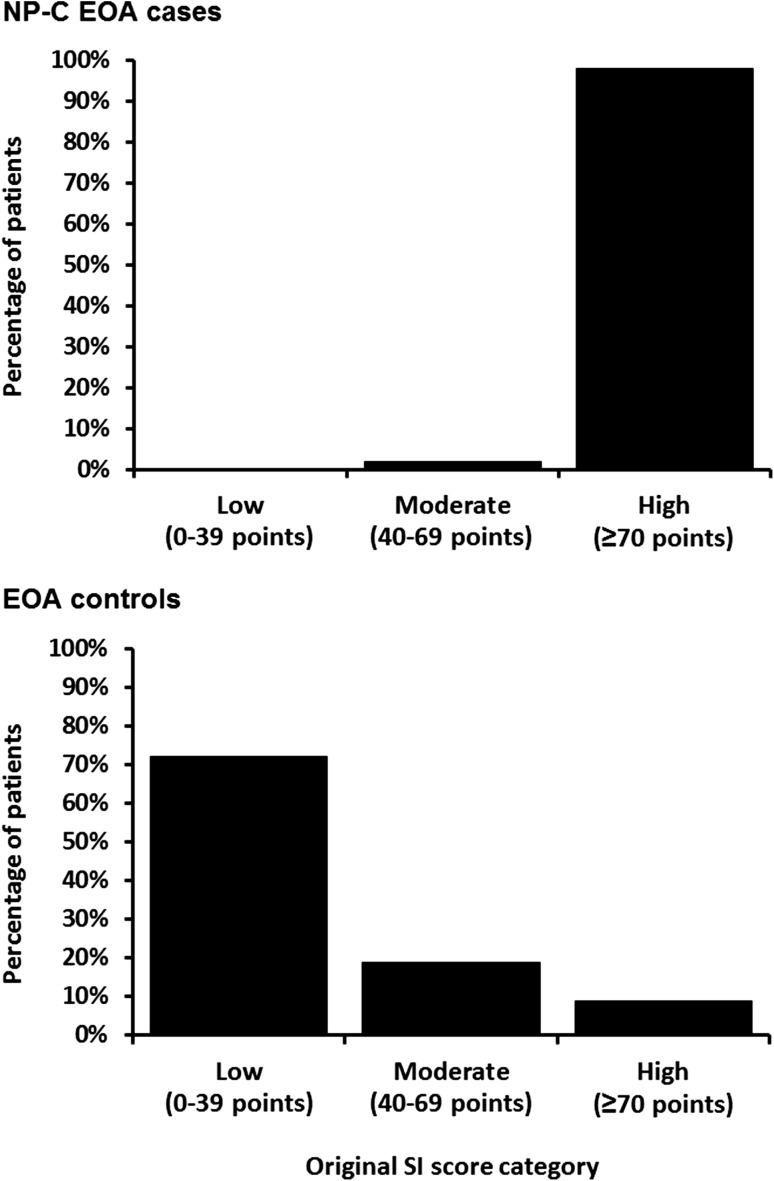
Proportions of NP-C EOA cases and EOA controls with low, moderate and high RPS on the original NP-C SI

Findings from the descriptive analysis of the proportions of patients with low (0–39 points), moderate (40–69 points) or high (≥70 points) RPS are summarised in Fig. 2. A notable number of EOA control patients had a moderate [*n* = 16/86 (19 %)] or high [*n* = 8/86 (9 %)] RPS. However, the scores of the NP-C EOA cases were significantly higher (*p* < 0.001, Fisher’s exact test), and most of them had a high RPS [46/47 (98 %)].

**Fig. 3:**
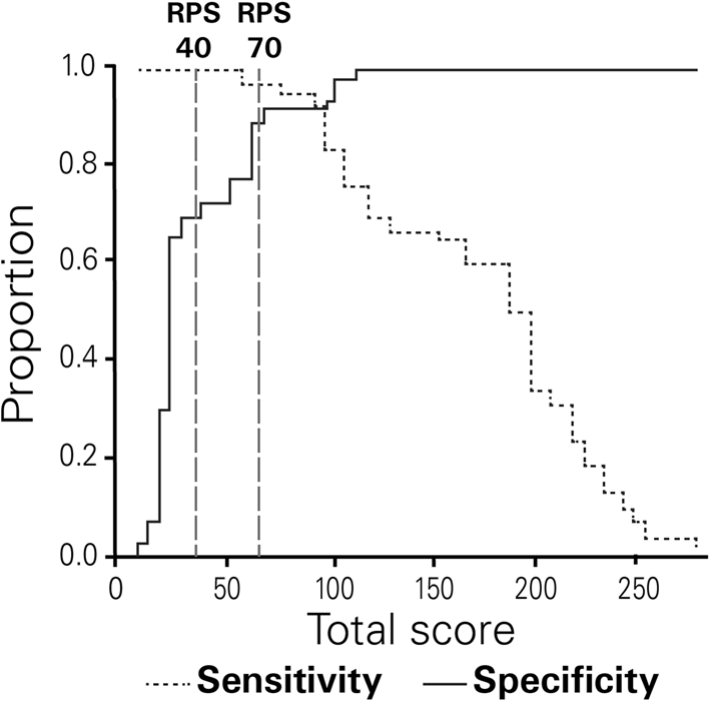
Sensitivity-specificity analysis for NP-C SI RPS in NP-C EOA cases versus EOA controls. *Dashed* *vertical* *lines* represent cut-off values for moderate (40) and high risk (70) of NP-C

Figure 3 illustrates findings from the analysis of RPS sensitivity and specificity in NP-C EOA cases versus EOA controls. Predictably, specificity increased and sensitivity decreased as total RPS rose. The cut-off RPS of 40 (for moderate suspicion) provided a sensitivity of 100 % and a specificity of 70 %. The cut-off RPS of 70 (for high suspicion) provided a sensitivity of 98 % and a specificity of 91 %.

Univariable linear regression modelling and ROC analysis of RPS revealed an AUC of 0.982 in NP-C EOA cases versus EOA controls (Supplementary Fig. S1), which compares well with findings from a post hoc subgroup analysis for patients aged ≥4 years who were included in the original SI development study (AUC 0.997) [15].

**Fig. 4:**
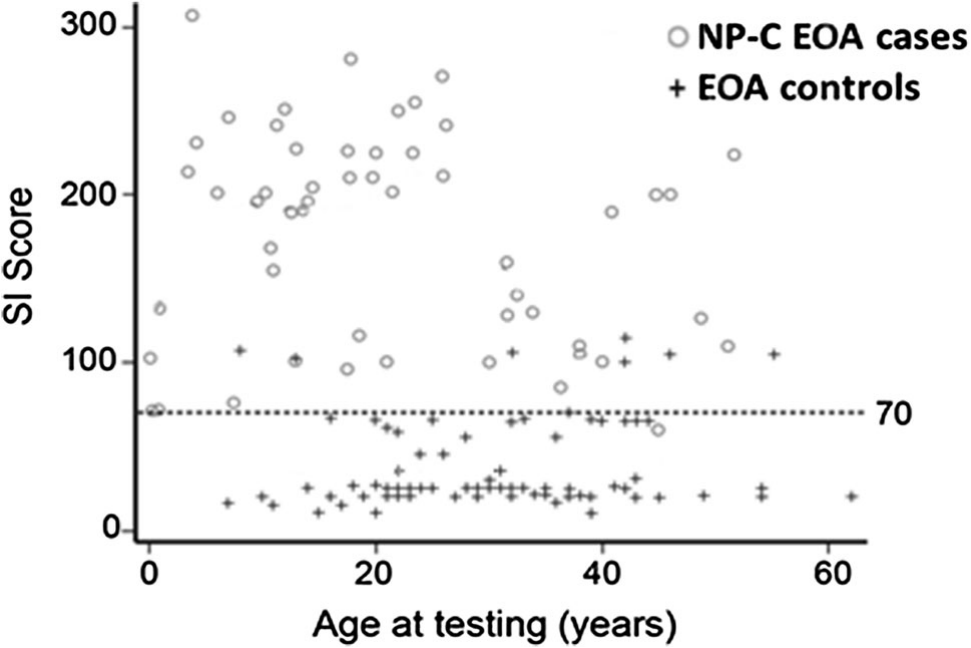
Total NP-C SI risk prediction scores by patient age

The discriminatory performance of the NP-C SI appeared unaffected by patient age (Fig. 4). Area under the curve values for subgroups of patients aged >4, >10 and >18 years were 0.982, 0.980 and 0.977, respectively, indicating excellent performance across all age groups.

### Assessment of 2/3 SI performance

Given that clinical routine often requires very rapid and efficient bedside testing, we evaluated a shorter form of the NP-C SI that required assessment of only three clinical features, called the ‘2 out of 3’ tool (‘2/3’ SI). From the panel of 21 original SI items, VSGP, pre-senile cognitive decline and/or dementia, dystonia, and splenomegaly were identified as those with the greatest sensitivity and specificity for the detection of NP-C (Table 2). For the 2/3 SI only the first three features were selected (VSGP, pre-senile cognitive decline and/or dementia, and dystonia).

**Table 2:** Findings from sensitivity and specificity analyses of the five NP-C SI key signs and symptoms providing the best performance based on co-occurrence with ataxia

Manifestation	Sensitivity	Specificity
VSGP (%)	94	92
Pre-senile cognitive decline and/or dementia (%)	94	77
Dystonia (%)	49	95
Isolated unexplained splenomegaly ± hepatomegaly (%)	47	100
Acquired and progressive spasticity (%)	32	42
Prolonged unexplained neonatal jaundice or cholestasis (%)	17	100
Hydrops foetalis	ND	ND
Siblings with foetal ascites	ND	ND
Gelastic cataplexy (%)	19	100
Dysarthria and/or dysphagia (%)	83	13
Hypotonia (%)	2	100
Delayed developmental milestones (%)	30	81
Seizure (partial or generalised) (%)	28	92
Myoclonus (%)	11	95
Acquired and progressive spasticity (%)	32	42
Psychotic symptoms (%)^a^	30	95
Treatment-resistant psychiatric disorders (%)	19	86
Disruptive/aggressive behaviour in adolescence and childhood (%)	6	100
Other psychiatric disorders (%)	19	93
Parent or sibling with NP-C (%)	13	100
Cousin with NP-C (%)	2	100

*ND* not determined (none of the EOA subjects had the sign or symptom)

^a^Hallucinations, delusions and/or thought disorder

**Fig. 5:**
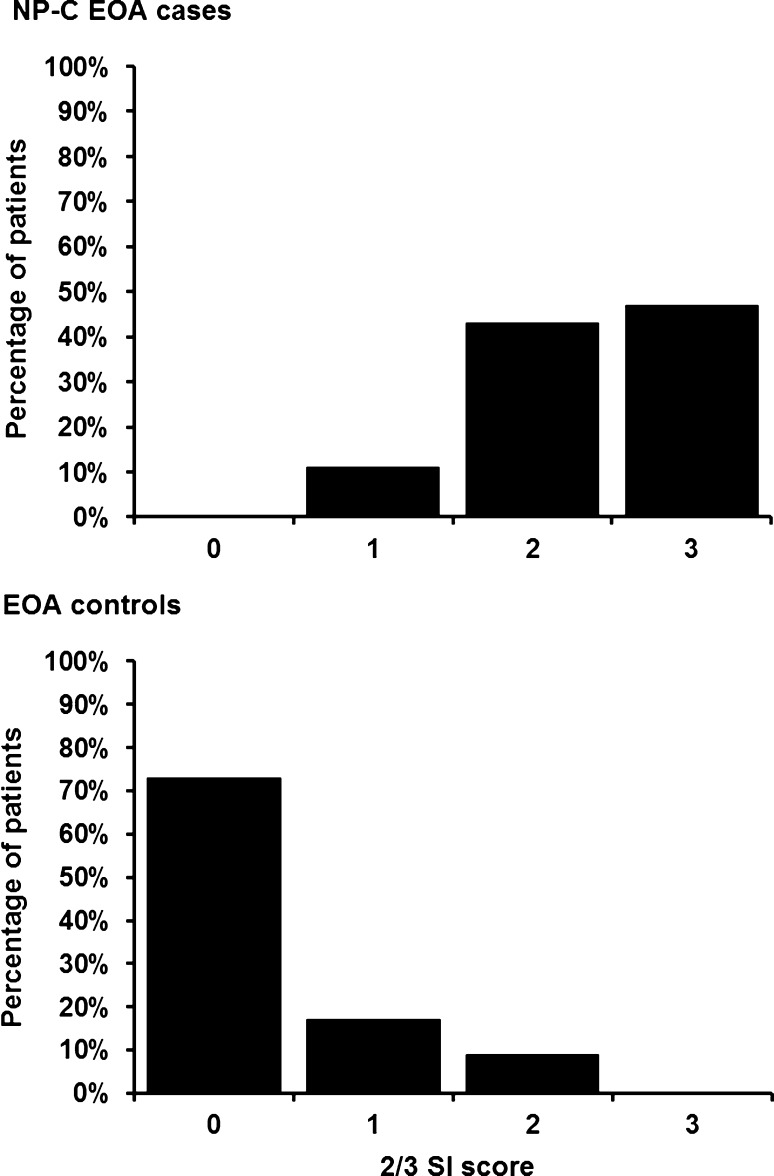
Distribution of 2/3 SI scores among NP-C EOA cases and EOA controls

We observed a clear difference in the distribution of 2/3 SI scores between NP-C EOA cases and EOA controls (Fig. 5). In total, 90 % of NP-C EOA cases had 2/3 SI scores of 2 or 3. In contrast, 90 % of EOA controls had scores of 0 or 1 on this tool. Based on ROC analyses, the 2/3 SI tool was effective at discriminating NP-C EOA cases from EOA controls, providing an AUC of 0.961 in NP-C EOA cases versus EOA controls, which is comparable with the excellent value achieved with the original SI tool in this ataxia cohort.

From sensitivity and specificity analyses at different 2/3 SI score levels, a score of 1 point (indicating moderate suspicion) provided a sensitivity of 100 % and a specificity of 73 %, and a score of 2 points (indicating high suspicion) provided a sensitivity of 89 % and a specificity of 91 %. A score of 3 points provided 100 % specificity: sensitivity at this level was relatively low (47 %).

Post hoc statistical testing, taking 2 points on the 2/3 tool and a score of 70 on the original SI as cut-off values indicating high suspicion of NP-C, indicated that the original SI had the greatest overall sensitivity (0.89 versus 0.98; *p* = 0.0078).

### Subgroup analysis excluding VSGP and Friedreich’s ataxia

We conducted subgroup analyses to assess the effect of absent VSGP on original SI and 2/3 SI performance, and the influence of Friedreich’s ataxia (FRDA) on the sensitivity and specificity of both SI scores. Predictably, the absence of VSGP rendered the 2/3 SI substantially less sensitive for the detection of NP-C, while the sensitivity of the original SI was relatively unaffected (Supplementary Fig. S2). The preponderance of FRDA in our cohort had no influence on the observed sensitivity or specificity of either the original SI or the 2/3 SI (Supplementary Fig. S2).

## Discussion

A large proportion of adult NP-C patients still remain undiagnosed in clinical practice [14]. The clinical recognition of NP-C is still affected by levels of awareness of the disease, and on the ability of diagnosing physicians to recognise suggestive signs and symptoms. The NP-C SI can aid in establishing a vital diagnostic link between clinical observations and eventual laboratory confirmation. However, while this tool is known to be effective in distinguishing NP-C patients from the general population [15], its ability to distinguish between NP-C cases and other multisystemic neurologic diseases with a high load of NPC-compatible disease features—such as EOA—has been questioned [4, 10]. Further, the identification of a clinical bedside tool that allows rapid appraisal of NP-C suspicion is of particular importance for EOA, as patients with this condition have been shown to represent a high-risk group for NP-C [12].

Excellent discriminatory performance in distinguishing EOA NP-C cases from EOA controls was observed with both the original NP-C SI and the novel 2/3 SI tool. The latter is easier to remember and quicker to apply than the original NP-C SI, and yet does not lose any discriminatory power. Based on the current findings we would recommend that, for the practical application of these SI tools, genetic testing for NP-C mutations should be considered advisable at thresholds of ≥70 points for the original SI and 2 or 3 points for the 2/3 tool.

Importantly, the EOA control group used for comparative reference with the NP-C EOA case group in this study comprised a wide range of EOA aetiologies, with incidences similar to those generally encountered in clinical practice [2, 5]. The proportion of patients in whom molecular diagnoses based on causal ataxia gene mutations were established (59 %) was similar to findings from previous studies in both European cohorts (55 %) [2] and high-consanguinity non-European cohorts (66 %) [5]. Further, as in the general EOA population [2], FRDA represented the bulk of genetically confirmed EOA cases (59 % of all genetically solved EOA cases) in our EOA control cohort. Subgroup analysis excluding FRDA patients showed equally good discriminatory performance of both original and 2/3 NP-C SI scores among complex non-FRDA EOA patients, where differential diagnosis is particularly complicated.

The notable numbers (approximately one-third) of EOA controls with moderate-to-high scores on the original NP-C SI confirm that EOA indeed has a high ‘background level’ of broadly NPC-compatible multisystemic disease features [3, 10]. All of the molecular EOA diagnoses identified among our EOA controls (see Supplementary Table 1) are known to present with a substantial extra-cerebellar disease load. This placed a high burden on the NPC-SI to provide sufficient discriminatory power to distinguish the NP-C cases.

Modelling assessments identified the top discriminatory features from the original SI tool for NP-C, from which VSGP, pre-senile cognitive decline and/or dementia, and dystonia were selected for incorporation into the 2/3 SI tool. The fact that VSGP and cognitive decline/dementia in particular were all recorded in >80 % of NP-C EOA cases confirms and extends findings from an earlier study [10]. Although splenomegaly yielded degrees of sensitivity and specificity for NP-C cases similar to that of dystonia, it was not selected for the 2/3 SI tool because it is frequently overlooked in clinical practice, is often mild or even absent in adult-onset cases [11], and is not always easy to evaluate on a mere clinical basis. Acquired and progressive spasticity was not selected based on its relatively low sensitivity and specificity, demonstrating that it is a frequent feature across many EOAs of different aetiologies [4].

Several limitations apply to this study. Firstly, our findings on the excellent discriminatory power of both the original and 2/3 NP-C SI in EOA are limited by the retrospective study design, which engender potential bias. Prospective studies are highly warranted to confirm the current findings. The 2/3 SI tool also requires further, independent validation in unrelated EOA cohorts. Other prospective studies will also help address the discriminatory power of the both SI tools in other patient populations with characteristic NP-C features, such as those with early onset cognitive decline or signs of organic, treatment-resistant psychosis.

All patients in this analysis were >4 years old. Previous studies have demonstrated that the discriminatory performance of the original NP-C SI is the greatest in non-infantile patients [6, 17]. Nevertheless, regression analysis in the current study to identify any relationship between patient age and original NP-C SI scores demonstrated the ability of this tool to distinguish between NP-C EOA and non-NP-C EOA across all patients aged >4 years. It should also be noted that the 2/3 SI is unlikely to be helpful in early infantile patients or neonates. While VSGP is a common neurological finding in patients with NP-C, particularly among those with juvenile- or adolescent/adult-onset neurological symptoms, it is less frequent among infantile-onset patients who are more commonly characterised by visceral manifestations. Our subgroup analyses revealed that the absence of VSGP rendered the 2/3 SI substantially less sensitive for the detection of NP-C: the sensitivity of the original SI was relatively unaffected. Published data indicate that the occurrence of VSGP is highly correlated with that of ataxia in NP-C [17], so if VSGP is not present it is likely that a patient would also not exhibit discernible ataxia, which would render the 2/3 even less effective. For these reasons, a separate SI tool targeted towards use in infantile patients is currently under development: initial findings from a total of 200 patients (106 NP-C infants and 94 non cases and controls) indicate improved discriminatory performance compared with the original SI [7].

Finally, while this study demonstrated that both the new 2/3 tool and the original SI provided equivalent areas under the ROC curve (both >0.95) and were effective in distinguishing between NP-C and non-NP-C EOA patients, the original NP-C SI demonstrated the greatest overall sensitivity compared with the 2/3 tool. Given the low prevalence of NP-C, as a rare disease, this might lead some clinicians to choose the original NP-C SI in preference to the 2/3 tool, despite the obvious advantages of the 2/3 tool in terms of rapidity and convenience.

## Conclusion

In conclusion, this study showed that the original SI has an excellent discriminatory performance even in multisystemic neurologic diseases with a high load of NPC-compatible disease features like EOA. In addition, the much simpler and more rapidly applicable 2/3 SI tool showed similar sensitivity and specificity to the original SI, and might serve as a more easily applicable tool for bedside assessment. While we would recommend application of the original SI in patients who do not have VSGP, the abbreviated 2/3 tool would seem ideally suited for initial bedside evaluations for NP-C and should also prove useful in broader screening studies in EOA patients.

## Electronic supplementary material

Below is the link to the electronic supplementary material.

**Supplementary Fig. S1** ROC curves for NP-C SI risk-prediction scores in NP-C EOA cases versus EOA controls (TIFF 410 kb)

**Supplementary Fig. S2** Sensitivity and specificity of original and 2/3 SI in subgroups of patients aged ≥ 4 years (TIFF 47 kb)

Supplementary material 3 (DOCX 39 kb)
