# Supplementary figures and images for: Identifying Niemann–Pick type C in early-onset ataxia: two quick clinical screening tools
Source: J Neurol. 2016 Jun 17;263(10):1911–8. doi: 10.1007/s00415-016-8178-0 (PMC5037150; doi:10.1007/s00415-016-8178-0)

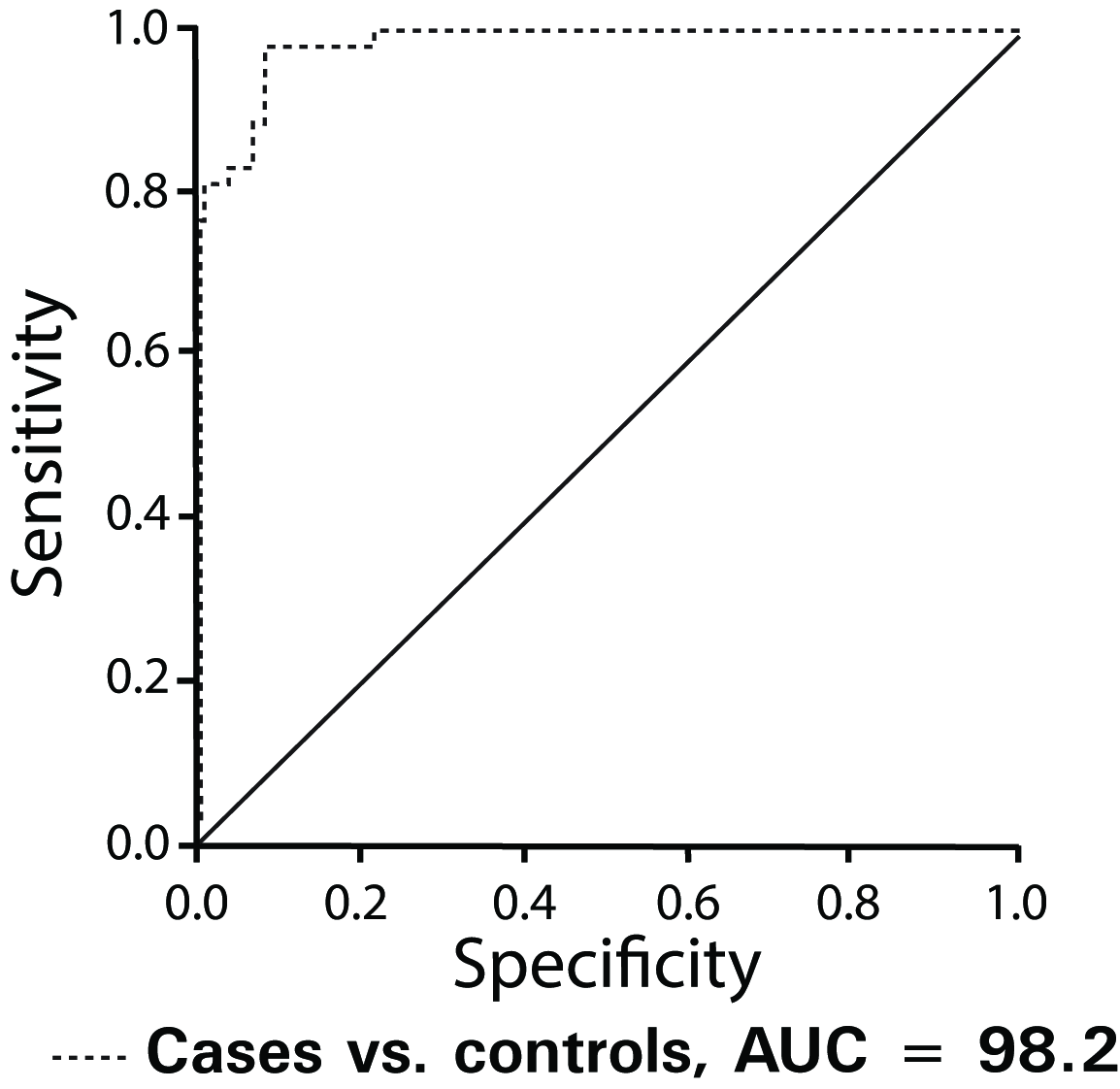

Supplement: Supplementary file 1 — Supplementary Fig. S1 ROC curves for NP-C SI risk-prediction scores in NP-C EOA cases versus EOA controls (TIFF 410 kb) [file 415_2016_8178_MOESM1_ESM.tif]

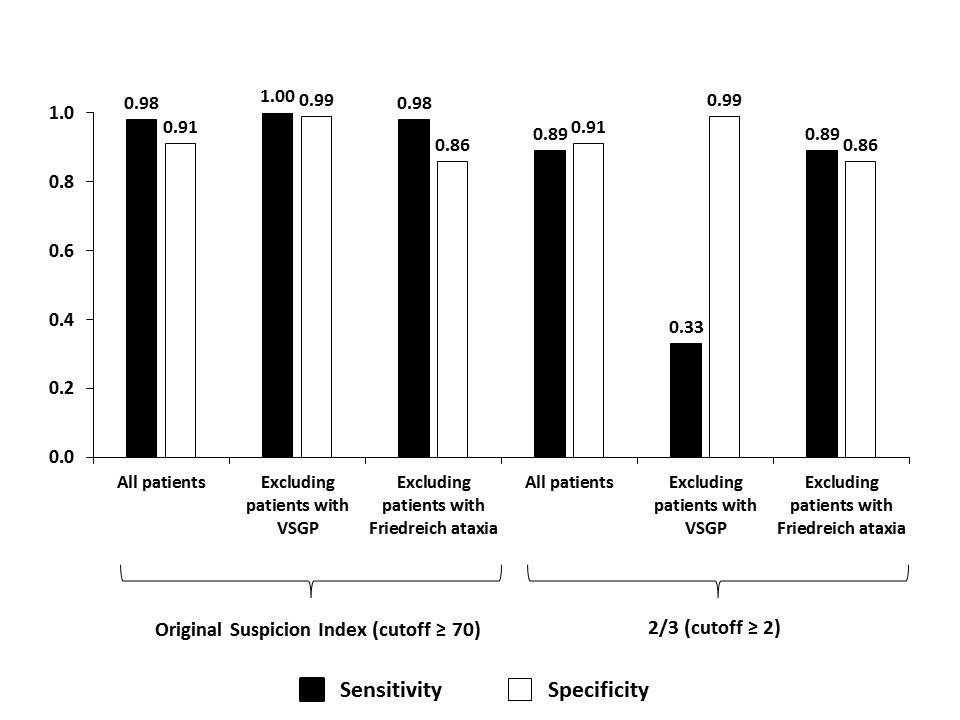

Supplement: Supplementary file 2 — Supplementary Fig. S2 Sensitivity and specificity of original and 2/3 SI in subgroups of patients aged ≥ 4 years (TIFF 47 kb) [file 415_2016_8178_MOESM2_ESM.tif]
