# Supplementary material for: Identifying Niemann–Pick type C in early-onset ataxia: two quick clinical screening tools
Source: J Neurol. 2016 Jun 17;263(10):1911–8. doi: 10.1007/s00415-016-8178-0 (PMC5037150; doi:10.1007/s00415-016-8178-0)
Supplement: Supplementary file 3 — Supplementary material 3 (DOCX 39 kb) [file 415_2016_8178_MOESM3_ESM.docx]

**Supplementary Table 1.** Clinical diagnoses in genetically confirmed EOA cases (N = 51 out of 86 EOA cases; total = 59.3%)

| **Genetic diagnosis** | **Disease  gene** | **Number (%) patients** |
| --- | --- | --- |
| Ataxia with oculomotor apraxia 1, EOAH, AOA1 | APTX | 1 (2.0)^a^ |
| Ataxia with oculomotor apraxia 2, SCAR1, AOA2 | SETX | 2 (3.9)^a^ |
| Spastic ataxia Charlevoix-Saguenay, ARSACS | SACS | 3 (5.9)^a^ |
| Ataxia telangiectasia, AT | ATM | 1 (2.0)^a^ |
| Familial isolated decificieny of vitamin E, VED | TTPA | 1 (2.0)^a^ |
| Cerebrotendinous xanthamatosis, CTX | CYP27A1 | 1 (2.0)^a^ |
| Friedereich ataxia, FRDA | FXN | 30 (58.8)^a^ |
| Boucher-Neuhauser-Syndrome BNHS | PNPLA6 | 1 (2.0)^a^ |
| Sensory ataxic neuropathy, dysarthria, and ophtalmoparesis, SANDO | POLG | 5 (9.8)^a^ |
| Spastic paraplegia 7, SPG7 | SPG7 | 2 (3.9)^a^ |
| Spinocerebellar ataxia 8, SCAR8 | SYNE1 | 1 (2.0)^a^ |
| Tay-Sachs disease, TSD | HEXA | 1 (2.0)^a^ |
| Other |  | 2 (3.9)^a^ |

^a^*Percentage calculated relative to number of patients with identified genetic mutation (N = 51).*
